# Copper(II) and Zinc(II) Complexes with Bacterial Prodigiosin Are Targeting Site III of Bovine Serum Albumin and Acting as DNA Minor Groove Binders

**DOI:** 10.3390/ijms25158395

**Published:** 2024-08-01

**Authors:** Lena Pantelic, Sanja Skaro Bogojevic, Tina P. Andrejević, Bojana V. Pantović, Violeta R. Marković, Darko P. Ašanin, Žiko Milanović, Tatjana Ilic-Tomic, Jasmina Nikodinovic-Runic, Biljana Đ. Glišić, Jelena Lazic

**Affiliations:** 1 Institute of Molecular Genetics and Genetic Engineering, University of Belgrade, Vojvode Stepe 444a, 11000 Belgrade, Serbia; lpantelic@imgge.bg.ac.rs (L.P.); sanja.bogojevic@imgge.bg.ac.rs (S.S.B.); tatjanait@imgge.bg.ac.rs (T.I.-T.); jasmina.nikodinovic@imgge.bg.ac.rs (J.N.-R.); 2 Department of Chemistry, Faculty of Science, University of Kragujevac, Radoja Domanovića 12, 34000 Kragujevac, Serbia; tina.andrejevic@pmf.kg.ac.rs (T.P.A.); bojana.pantovic@pmf.kg.ac.rs (B.V.P.); violeta.markovic@pmf.kg.ac.rs (V.R.M.); 3 Department of Science, Institute for Information Technologies Kragujevac, University of Kragujevac, Jovana Cvijića bb, 34000 Kragujevac, Serbia; darko.asanin@uni.kg.ac.rs (D.P.A.); ziko.milanovic@uni.kg.ac.rs (Ž.M.)

**Keywords:** bio-refinery, waste, *Serratia marcescens*, prodigiosin, metal complexation, protein interactions, DNA interactions

## Abstract

The negative environmental and social impacts of food waste accumulation can be mitigated by utilizing bio-refineries’ approach where food waste is revalorized into high-value products, such as prodigiosin (PG), using microbial bioprocesses. The diverse biological activities of PG position it as a promising compound, but its high production cost and promiscuous bioactivity hinder its wide application. Metal ions can modulate the electronic properties of organic molecules, leading to novel mechanisms of action and increased target potency, while metal complex formation can improve the stability, solubility and bioavailability of the parent compound. The objectives of this study were optimizing PG production through bacterial fermentation using food waste, allowing good quantities of the pure natural product for further synthesizing and evaluating copper(II) and zinc(II) complexes with it. Their antimicrobial and anticancer activities were assessed, and their binding affinity toward biologically important molecules, bovine serum albumin (BSA) and DNA was investigated by fluorescence emission spectroscopy and molecular docking. The yield of 83.1 mg/L of pure PG was obtained when processed meat waste at 18 g/L was utilized as the sole fermentation substrate. The obtained complexes CuPG and ZnPG showed high binding affinity towards target site III of BSA, and molecular docking simulations highlighted the affinity of the compounds for DNA minor grooves.

## 1. Introduction

A staggering amount of food is wasted across the production-to-consumption chain annually while the world is fighting hunger and the environmental impact of waste [1,2]. Recognizing the urgency of the situation, there is a growing need for the valorization of waste streams to create sustainable solutions within a circular economy framework [3]. Integrating waste into bioprocesses for the production of valuable bio-products, often referred to as ‘bio-refining’, has emerged as a promising approach [4]. Utilizing the metabolism of microorganisms represents a sustainable way of converting food waste into multifunctional bio-products with high value, such as bacterial biopigments, as they have shown potential for various applications in food, pharmaceutical and textile industries [5].

Prodigiosin (PG), a red natural product synthesized primarily by *Serratia marcescens*, manifests a plethora of biological activities, including pro-apoptotic [6,7] and antimicrobial activities [8], positioning it as a promising bioactive compound which can be obtained through microbial fermentation [9]. Its application is hindered due to its high commercial cost, so considerable efforts have been made to lower the production costs of PG, mostly relying on bio-refining, as well as agro-industrial waste [10] and chemical industry waste [11]. While PG demonstrates significant antiproliferative activity towards various cancers, including lung [12], breast [13,14], colon [15] and liver [16], its targets are diverse and its selectivity is often low [17], which is why it has not advanced in anticancer clinical trials.

PG emerged as an attractive parent molecule for the development of alternative bioactive compounds with higher selectivity [18]. While significant progress has been made, further research is warranted to explore its modification in order to enable clinical and practical applications. One of the most effective strategies for improvement and/or modification of the biological activities of the organic compounds is their complexation with both essential and non-essential metal ions [19,20]. In comparison with purely organic compounds, which possess one- or two-dimensional shapes, different geometries and predominantly three-dimensional shapes have been observed for metal complexes. Since the molecular shape is an important factor in molecular recognition by the biomolecule, these characteristics of metal complexes contribute to their better therapeutic profile in respect to purely organic compounds [21,22,23,24,25,26]. Besides that, metal complexes can manifest their activities through different pathways, such as redox activation, ligand release or the exchange and catalytic production of reactive oxygen species [27,28]. As a proof of the concept, the Community for Open Antimicrobial Drug Discovery (CO-ADD) evaluated the antimicrobial properties of 906 metal-containing compounds and found out that these compounds have a significantly higher success rate (9.9%) in comparison with the organic compounds (0.87%) in the CO-ADD database [29]. Thus, 88 metal compounds have shown activity against at least one of the evaluated microbial strains, while being non-toxic on the normal mammalian cell lines and not displaying hemolytic properties [29]. Moreover, many recent studies have highlighted that the combination of different metal ions, including zinc(II), copper(II), silver(I), gold(III), ruthenium(II/III) and rhenium(I), with clinically used antifungal drugs has led to their improved activity, uptake and bioavailability [19,30,31,32,33,34,35]. This strategy can be also used in the design of novel anticancer, antibacterial and anti-parasitic agents [20].

Taking the above-mentioned facts into account, in this study, bacterial PG was produced in a sustainable manner from food waste and used for the complexation of copper(II) and zinc(II) ions, leading to the formation of CuPG and ZnPG complexes, respectively, which were tested as antimicrobial and anticancer agents. The interaction between these metal complexes and bovine serum albumin (BSA) was evaluated to comprehend their capacity as drug delivery systems, given that BSA is a significant protein carrier in circulation. Additionally, exploring their binding to DNA provided insights into their mechanism of action at the molecular level, which is essential for the design of new antimicrobial and anticancer agents. In summary, this approach offered a promising solution to food waste management and the production of PG at lower costs and highlighted the promising therapeutic potential of PG and its complexes.

## 2. Results and Discussion

### 2.1. PG Production Optimization Using Waste Streams

Food waste often possesses the potential to be upcycled and transformed into valuable bio-products, such as PG. In this work, we substituted the commercially used bacteriological media components with different waste materials, thus making the production process more economically viable. In order to identify a suitable waste stream for PG production, Fermentation Broth (FB) was selected as the control medium and the basis for formulating 36 alternative media in six different waste streams, including potato peel (PP), stale bread (SB), mixed waste (MW), yogurt (YO), peeled boiled eggs (BE) and processed meat (PM) (Table S1).

**Figure 1:**
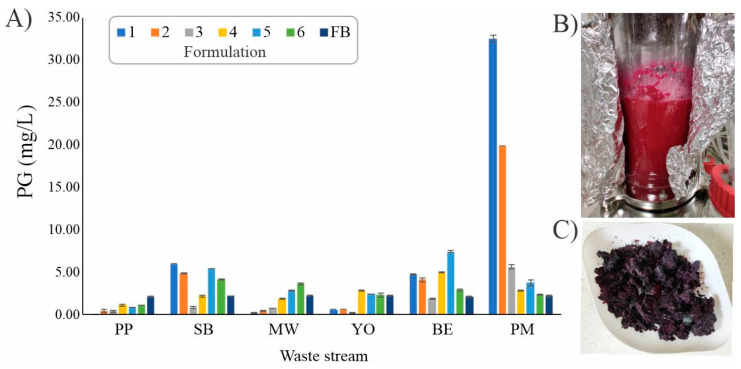
(**A**) The influence of various waste streams and their formulations on PG production in *S. marcescens* ATCC 27117: potato peel (PP), stale bread (SB), mixed waste (MW), yogurt (YO), peeled boiled eggs (BE), processed meat (PM); (**B**) the bioreactor at the end of the PM-1 fermentation; (**C**) lyophillized *S. marcescens* ATCC 27117 cells.

The highest rate of PG production, at 32.52 mg/L, was recorded in PM-1 medium, with sole utilization of PM at 18 g/L. The next best result at 19.87 mg/L was observed in PM-2, consisting of 18 g/L of PM and NaCl, KCl and MgSO_4_ salts (Table S1 and Figure 1). Statistical analysis demonstrated significant differences in PG production among the tested PM media. Notably, the PG production of 32.52 mg/L in PM-1 is 14.7-fold higher than the production of 2.21 mg/L of PG in FB medium (Figure 1).

In microbial processes, the elemental composition of the substrate and the balance between elements, especially the C/N ratio, has a significant influence on how microorganisms utilize organic materials, which was also shown for PG production [36,37]. Previously reported elemental analysis of waste materials [38] was analyzed to better understand the connection between PG production and substrate utilization. The elemental composition of ME revealed high carbon content at 50.73%, followed by nitrogen at 11.51%, with a C/N ratio of 4.4. Literature results suggest that the optimal C/N ratio for PG production ranges from 1.5 [37] to 10.0 [39] with lower values being more prominent [36]. However, the C/N ratio alone could not account for the overall differences in PG yields between the different waste-based media. The type of substrate, especially the complexity of the carbon source [40], plays a vital role in PG production. The initiation of 2-methyl-3-pentyl-pyrrole (MPP) synthesis, a precursor of PG in biosynthesis, begins with 2-octenal, primarily derived from the oxidation of fatty acids [9]. Therefore, adding certain fatty acid oils can enhance the synthesis of 2-octenal and MPP, consequently boosting PG production, an approach that has already been implemented in some studies [41]. Considering this, it should be noted that the high fat content found in PM could have played a role in enhanced PG production.

### 2.2. PG Production and Purification in the Bioreactor

To acquire a sufficient biopigment amount for metal complexation experiments, the cultivation of the producing strain was scaled up in the 4.5 L bioreactor, under predefined conditions (pH 7.0, 28 °C/301.15 K, 500 rpm). Fermentations were carried out in 3 L of FB and PM-1 media. PG concentration was monitored spectrophotometrically [42], and the yields were determined after extraction and chromatographic purification, where 83.1 ± 3.0 mg/L of PG was produced in PM-1 and 6.2-fold lower production of 13.3 ± 1.1 mg/L PG was obtained from FB. Notably, the scaled-up fermentation in PM-1 yielded 2.6-fold higher PG production compared to the flask optimization experiment (Figure 1), where PG production was at 32.52 mg/L.

The use of ram horn, a byproduct of the meat industry, was explored as a media component in a submerged culture of the local isolate *S. marcescens* MO-1 [43]. The highest yield of 277.74 mg/L PG was achieved using a medium containing 0.4% yeast extract and 1% mannitol, with the addition of 0.4% (*w*/*v*) ram horn peptone at pH 7 after 48 h. That yield is 3.3-fold higher in comparison to this study, but in this work PG was produced solely using the waste stream with no commercial components in the medium. Furthermore, MO-1 strain was isolated from fields contaminated with pesticides [43], where PG biosynthesis plays an eco-physiological role for microbial survival, while ATCC 27117 is a commercial strain. The selection of the high-producing strain is key to maximizing PG yield [9], so there is potential for method refinement by exploring alternative high-producing wild-type or genetically modified strains. Prodigiosin-like pigment produced by *S. marcescens* SMΔR strain, a deletion mutant of *S. marcescens* SS-1, was obtained from media enriched by different plant-derived oils. Adding 6% (*v*/*v*) sunflower oil to the modified LB medium (13.4 g/L yeast extract and 22.5 g/L tryptone) afforded 790 mg/L of prodigiosin-like pigment, while 4% olive oil and 4% soybean oil yielded 579 and 525 mg/L, respectively [41]. Once again, their yields are more favorable in comparison to this study, but the obtained prodigiosin-like pigment was shown to be undecylprodigiosin, which does belong to the family of prodiginines, but is not PG itself. It should also be noted that the fermentation time plays an important role when estimating the economic feasibility of a fermentative process [44] and the fermentation time in the above-mentioned studies was 3- to 5-fold longer in comparison to 12 h accomplished in this study.

Previous research has demonstrated the successful utilization of waste as a substrate for PG production in *S. marcescens* through solid-state fermentation, where tannery fleshing and wheat bran were combined in a 7/3 ratio, supplemented with various salts (NaCl, KH_2_PO_4_, Na_2_HPO_4_, NH_4_Cl), resulting in a yield of 70.4 mg PG per 1 g of tannery fleshing after 82 h [45]. An even higher yield of 900 mg/L PG was isolated from *S. marcescens* ATCC 13880 grown on a solid medium containing 2% sucrose (carbon source), 1% casein hydrolysate (nitrogen source) and 4% peanut oil seed cake (sludge after oil extraction) as the fatty acid source at pH 7.5 after 72 h [46]. It was also shown that the incorporation of salts into the waste-based production media can enhance PG production [45,47]. Additionally, fed-batch fermentations for PG production were identified as optimal, as well as other biopigments [9,37,48], so these directions should be explored in the future.

### 2.3. Copper(II) and Zinc(II) Complexes with PG

**Figure 2:**
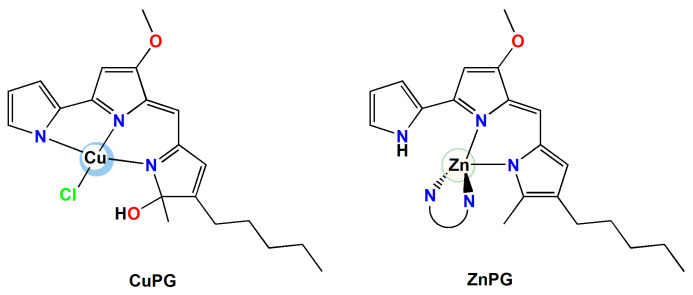
Structural formulae of copper(II) and zinc(II) complexes with PG [49].

In the present study, PG was used for the complexation of copper(II) and zinc(II) metal ions in accordance with the published method (Figure 2) [49]. The synthesized CuPG and ZnPG complexes are soluble in DMSO, DMF, methanol, acetonitrile and chloroform. They also remain coordinated to the metal ion in DMSO.

### 2.4. Antimicrobial Activity—Disc Diffusion Assay

Both PG and its metal complexes, CuPG and ZnPG, exhibited no antibacterial or antifungal activity against the tested strains (Figure S1). Low PG activity against *E. coli* and *P. aeruginosa* is in line with previous results, where these strains were shown to be resistant to PG [50]. It was previously observed that the antimicrobial activity of its copper(II) complexes is not the consequence of the metal ion [51], so CuPG complex also showed no antimicrobial activity. The absence of antibacterial activity in CuPG and ZnPG complexes can be ascribed to many factors. The arrangement of these complexes may impede their interaction with microbial targets, hence diminishing their effectiveness. The presence of metal ions might induce changes in the lipophilicity and overall charge distribution, impacting the capability of the complexes to penetrate microbial cell membranes. Finally, the environment around the metal centers could alter the binding affinity to critical microbial enzymes or DNA, thus limiting their antimicrobial potential. Further studies focusing on structural modifications and alternative metal ions may be necessary to improve the antimicrobial activity of PG-containing metal complexes.

### 2.5. Antiproliferative Activity

PG exhibited a high cytotoxicity of 3.71 µM against the healthy cells (MRC-5), which could be a contributing factor preventing PG from advancing into clinical practice so far, but it demonstrated good activity against both tested cancer cell lines (Table S2) [52]. In line with our results, previous studies of PG recognized its anticancer activity against A549, ranging from 0.09 μM [17] to 10 μM [17]. PG activity against HCT116 was reported at 0.12 μM following a 72 h treatment [53], which is 18-fold lower compared to our results after 48 h treatment. This highlights the necessity for developing derivatives of PG such as metal complexes with enhanced selectivity to address this issue. Both CuPG and ZnPG complexes exhibited significantly lower cytotoxicity against MRC-5, at 91.45 and 42.24 µM, respectively, but their anticancer potential was inferior to that of PG (Table S2). The reduced anticancer activity of these complexes might be a consequence of the alterations in the electronic properties and structural configuration upon metal coordination, which could affect their ability to interact effectively with essential cellular targets in cancer cells. Future research should focus on fine-tuning the structures of metal complexes to enhance their therapeutic efficacy, while maintaining low toxicity to healthy cells.

### 2.6. BSA Binding Study

**Figure 3:**
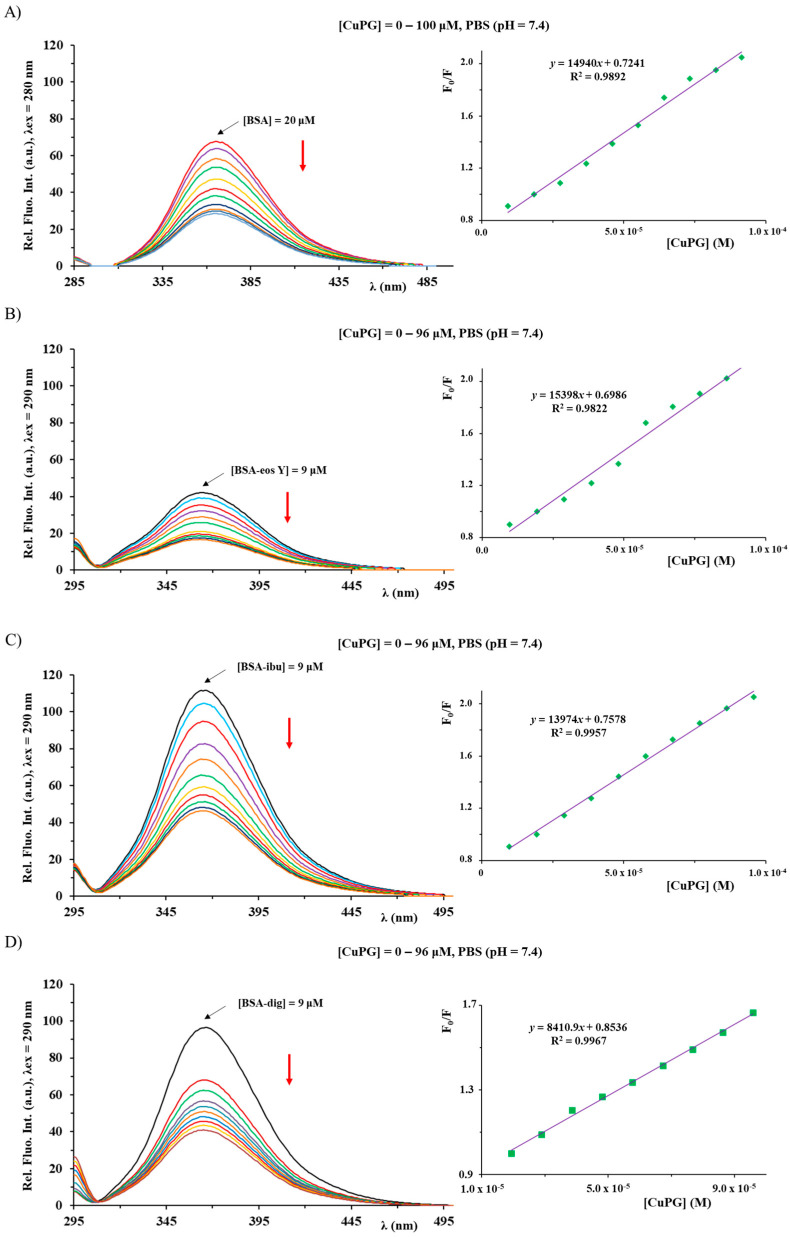
BSA fluorescence emission spectra in the presence of an increasing concentration of CuPG (**A**) and in the presence of the site markers (**B**–**D**). The red arrow shows the changes in the intensity after the addition of the complex. The inserted graph presents the F_0_/F dependence of complex concentration.

Considering that serum proteins have an important role for transporting various compounds to their targets [54], the interactions of PG and its copper(II) and zinc(II) complexes with BSA were initially investigated by florescence emission spectroscopy [55]. The addition of CuPG and ZnPG complexes and PG to a BSA solution of constant concentration (Figure 3 and Figures S2 and S3) led to the quenching of fluorescence of BSA at *λ* = 366 nm in a concentration-dependent manner, indicating their interaction with the studied protein. The measured fluorescence intensities were further analyzed by the Stern–Volmer and Scatchard equations (Table 1).

The presented *K*_A_ values show that the binding affinity of CuPG is higher than the affinity of ZnPG and PG; nevertheless, all the investigated compounds have revealed moderate affinity toward the studied BSA protein [56] with the *n* number being approximately 1. The *K*_q_ values are greater than the maximum diffusion constant of the biomolecule (2 × 10^10^ M^−1^s^−1^), being in accord with static quenching predominance and the forming of BSA adducts of PG and its investigated metal complexes [57]. The binding of PG to BSA with the change in the conformation and microenvironment of this protein was also previously confirmed by FT-IR, fluorescence, UV–Vis absorption and circular dichroism methods [58]. Moreover, PG could bind human serum albumin and bovine β-lactoglobulin, causing a strong fluorescence quenching of these proteins via the mechanism of static quenching [59].

The structure of BSA can be categorized into three domains that are homologous to each other. These domains are referred to as I (amino acid, AA residues 1–195), II (AA residues 196–383) and III (AA residues 384–583). It is worth noting that each of these domains contains subdomains A and B [60]. The presence of hydrophobic pockets inside IIA and IIIA subdomains, referred to as site I and site II, respectively, has been identified as the location of the binding sites for various aromatic compounds and heterocycles [61,62]. The identification of a third binding pocket located inside subdomain IB, also known as active site III, has recently been reported [63]. This binding site was determined to be the principal site for the binding of various chemicals such as bilirubin, hemin and sulfonamide derivatives. Available literature data indicate that various organometallic compounds show affinity towards this active site [56]. As part of this research, we assessed the affinity of the studied compounds (PG, CuPG and ZnPG) towards each of the three active sites of the BSA protein by a competitive fluorescence spectroscopy study, using eosin Y (eos Y), ibuprofen (ibu) and digitoxin (dig) as markers for sites I, II and III, respectively, and molecular docking analysis.

The studied protein and the site markers were mixed in equimolar concentrations (9 μM), and the investigated compounds were added in increasing concentrations (Figure 3 and Figures S2 and S3). If the studied compound and the corresponding marker bind to the same site, after addition to the BSA–marker solution, a compound will compete with the marker to bind to BSA, leading to a considerable change in the value of the binding constant in comparison to that in the absence of the marker [64]. The Stern–Volmer and Scatchard equations were used to analyze the obtained emission quenching data (Table 1). Based on these data, it can be seen that the largest decrease in the BSA binding constants of CuPG and ZnPG complexes is observable in the presence of the dig marker, indicating that they could compete with it to bind to site III. In addition, the slight changes in the *K*_A_ values for CuPG could also be noted in the presence of the other two markers, and to clarify this, we have decided to perform a molecular docking study. Also, an increase in the *K*_A_ values for the BSA binding of PG occurred in the presence of all three studied site markers, being in accordance with the non-competitive interference between PG and the site markers, suggesting that the compounds may cause structural changes in BSA, which can additionally generate more binding sites or enhance the accessibility of the existing binding sites, thereby increasing the binding affinity of the compound to the model protein [65].

**Figure 4:**
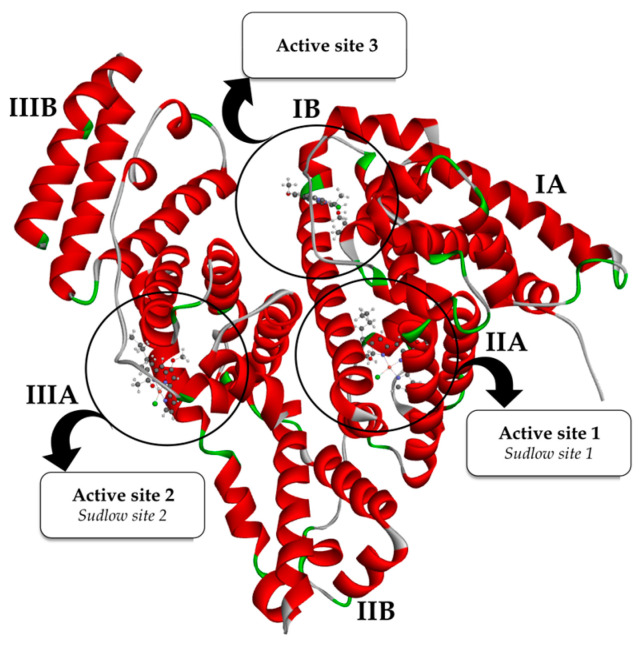
A three-dimensional representation of the most stable conformations of the CuPG complex bound to three different active sites (I (*subdomain IIA*), II (*subdomain IIIA*), III (*subdomain IB*)) of BSA (PDB: **4F5S**).

The outcomes derived from the molecular docking analysis for BSA interactions of PG, CuPG and ZnPG are provided in Table 2. The most stable conformations of the CuPG complex within different active sites of BSA are presented in Figure 4.

From the results in Table 2, it can be concluded that the binding activity/affinity of investigated complexes follows a descending order: BSA-III > BSA-II > BSA-I. This indicates that CuPG and ZnPG complexes exhibited the greatest affinity towards active site III. The conclusion above aligns with the experimentally determined *K*_A_ values (Table 1). ZnPG exhibited a higher affinity towards every active site that was examined as compared to CuPG. The observed outcome was expected because of the larger number of aromatic rings and increased affinity towards the hydrophobic pockets of BSA. The disparity in the reactivity of the examined complexes acquired through experimental and theoretical approaches can be ascribed to various factors. Water and solvation effects significantly impact the interaction between proteins and investigated compounds in experimental conditions, but these effects are not taken into account in docking studies. Furthermore, the existence of Cl atoms in the CuPG complex could potentially enhance specific ionic interactions and induce conformational alterations in BSA, which can be more accurately identified through experimental techniques. Experimental studies may exhibit variations in results due to the influence of laboratory conditions, such as pH, ionic strength and the presence of other molecules, which can selectively promote the reactivity of one complex over another.

PG exhibits a similar or higher affinity towards all active sites compared to the investigated complexes (Table 2). The better accommodation of PG in the BSA is a result of the existence of protonated pyrrole rings that establish additional interactions, together with the involvement of van der Waals contacts with AA residues, which can be seen from the higher values of ΔG*_vdw+hbond+desolv_* (Table 2). The distinction between theoretical and experimental values arises because docking studies prioritize less intricate molecules, such as free PG, due to their adaptability and effortless interactions with the target protein, which are facilitated by idealized conditions and static models. On the other hand, spectrofluorimetric experiments directly assess the interactions occurring in water-based solutions, taking into account the influence of water, ions and other molecules on the stability and strength of the binding. Metal complexes have specific coordination structures that improve their stability and specificity of binding to BSA as a consequence of the reaction medium, which is not taken into account in docking simulations.

**Figure 5:**
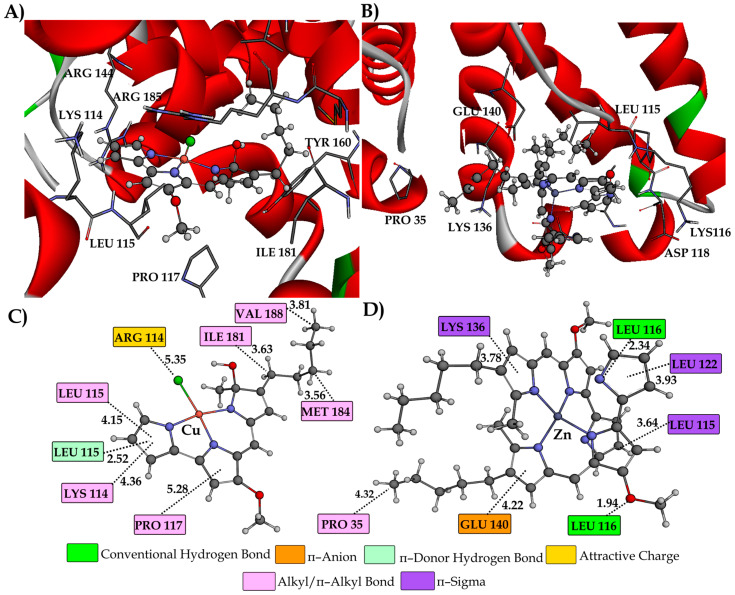
The most stable docking positions of CuPG (**A**) and ZnPG (**B**) in subdomain IB (active site III) of BSA (PDB code: **4F5S**) are represented in three dimensions. The complexes are shown as gray sticks for carbon atoms, with other atoms colored differently: nitrogen (N) in blue, oxygen (O) in red, chlorine (Cl) in green, copper (Cu) in pink and zinc (Zn) in violet. To enhance clarity, the remaining protein structure is not presented. The two-dimensional representations illustrate the interactions between CuPG (**C**) and ZnPG (**D**) and BSA, including interatomic distances (Å) from the molecular docking study. Different colors in the ZnPG interaction diagram represent various types of interactions, as indicated in the legend.

Figures S4 and S5 show the most stable conformations of the examined compounds within the hydrophobic pockets of active sites I and II, respectively. The conformations of the ZnPG and CuPG within active site III are illustrated in Figure 5. Furthermore, Figures S6 and S7 illustrate the most stable configurations of PG at different active sites of BSA, together with specific interactions with AA residues. The primary interaction between the investigated CuPG and ZnPG complexes and AA residues is through hydrophobic contacts, consistent with their structural properties.

In active site I (Figure S4), the investigated complexes establish one conventional hydrogen bond with AA residues ARG 256 (2.17 Å) and LYS 232 (1.88 Å) through the oxygen of the methoxy group. Additionally, residue LEU 237 (3.52 Å) forms a characteristic hydrophobic *π*–sigma interaction with the aromatic ring of the CuPG complex. AA residue ASP 236 establishes a π–sigma (3.68 and 3.61 Å) and a π–anion (3.07 Å) with the aromatic rings of the ZnPG complex. AA residues TYR 149, LYS 211, LEU 218, LYS 239, LEU 259 and ALA 290 establish *π*–alkyl interactions with aromatic and aliphatic parts of the investigated compounds. AAs ARG 198 (4.48 Å) and ARG 217 (3.52 Å) form π–cation interactions with the heterocyclic pyrrole ring of PG, whereas TRP 213 and LEU 346 form π–alkyl/alkyl interactions (Figure S7).

The CuPG molecule in active site II was stabilized through the formation of conventional hydrogen bonds with the AA residues SER 488 (2.11 Å) and ASN 390 (2.08 Å). PG establishes conventional hydrogen bonds with AA residues LEU 386 (2.84 Å) and ASN 390 (1.95 Å) by interacting with the –N and –NH groups of the pyrrole ring (Figure S7). The ZnPG complex forms a π–donor hydrogen bond with the –NH group of GLN 389 at a distance of 2.39 Å. Additionally, it forms a carbon–hydrogen bond with the oxygen atom of LEU 490, with a bond length of 3.46 Å. AA residues in hydrophobic pockets, LEU 259, PRO 383, LEU 386, ARG 409 and PRO 492, provided stability support by forming hydrophobic *π*–alkyl and alkyl bonds with the aromatic and aliphatic parts of the examined compounds.

As in previous cases, the results of the molecular docking study depicted in Figure 5 demonstrated that the most stable conformations of the tested complexes are largely stabilized by hydrophobic contacts. The AA residue LEU 116 forms a characteristic bifurcated conventional hydrogen bond with the polar nitrogen atom of the pyrrole ring (2.34 Å) and the oxygen –OCH_3_ group (1.94 Å) of the ZnPG complex. In addition, the AA TYR 160, through the oxygen of the carbonyl group, establishes a hydrogen bond (2.02 Å) with the hydrogen of the pyrrole ring of PG (Figure S7). The stabilization of the investigated compounds for active site III was also attributed to the presence of electrostatic interactions. The chlorine atom of the CuPG complex exhibited a partial negative charge and established an attractive charge contact with the AA residue ARG 114 (5.35 Å). In addition, it can be observed that GLU 140 forms a π–anion interaction (4.22 Å) with the aromatic ring of ZnPG. In this instance, the presence of hydrophobic interactions was the primary factor that significantly contributed to the overall stability of CuPG and ZnPG within active site III. The aromatic rings of ZnPG form π–sigma interactions with the AA residues LEU 115 (3.64 Å), LEU 122 (3.93 Å) and LYS 136 (3.78 Å). In addition, the stabilization of CuPG occurs through the involvement of AA residues, including LYS 114, LEU 115, PRO 117, ILE 181, MET 184 and VAL 188, which establish distinctive π–alkyl and alkyl interactions.

### 2.7. ct-DNA Binding Study

**Figure 6:**
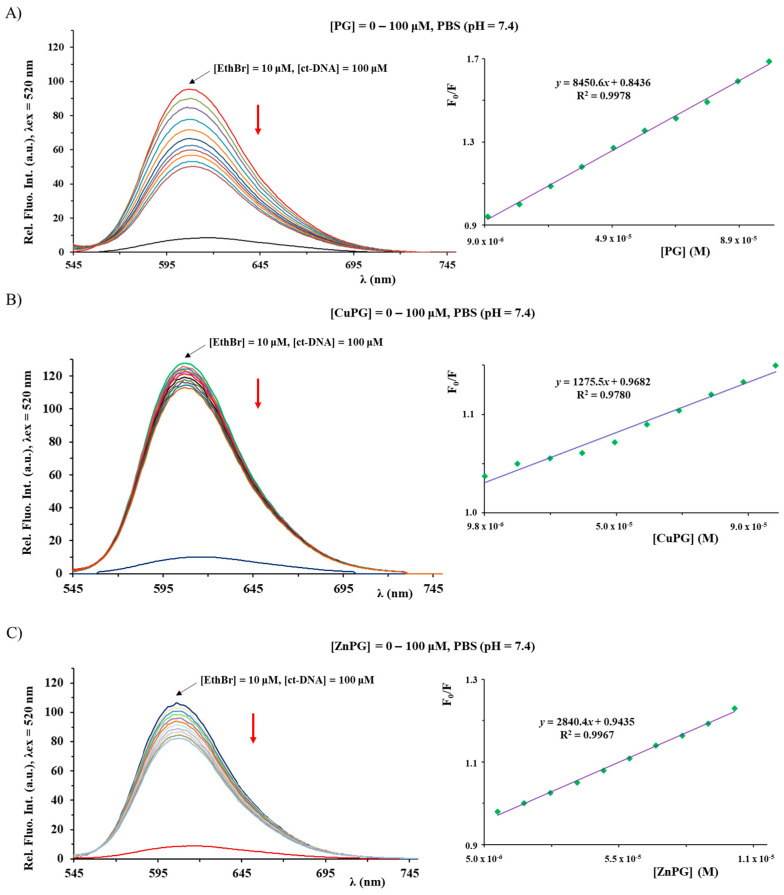
Fluorescence emission spectra of the ct-DNA–EthBr system in the presence of increasing concentrations of PG (**A**), CuPG (**B**) and ZnPG (**C**). The red arrow shows the changes in the intensity upon addition of the studied compound. The inserted graph represents the Stern–Volmer plot of the F_0_/F vs. [compound].

**Figure 7:**
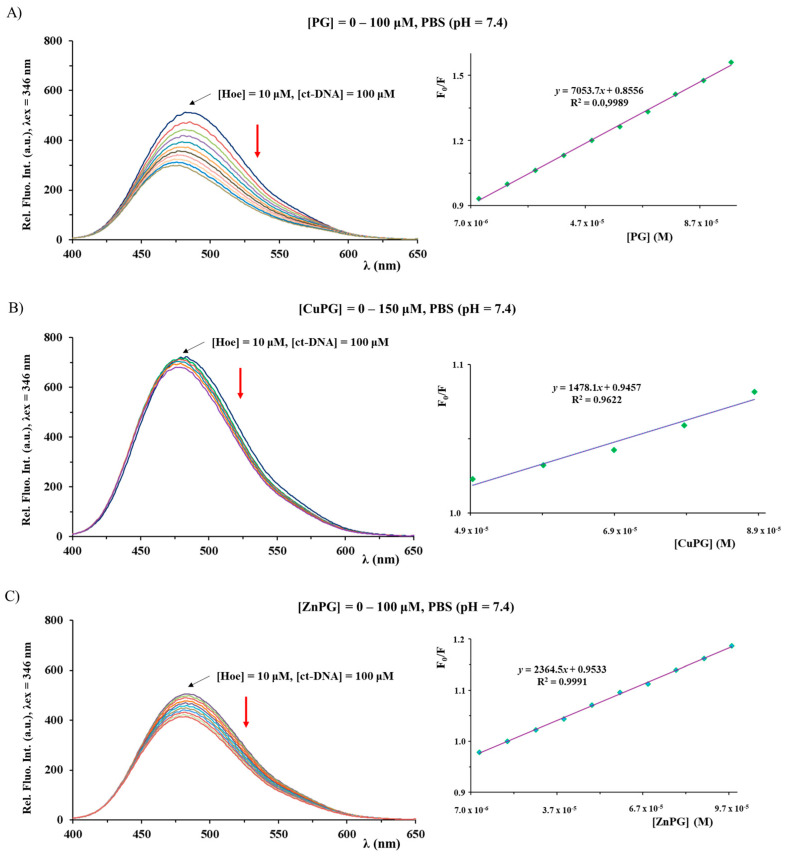
Fluorescence emission spectra for the ct-DNA–Hoe system in the presence of increasing concentrations of PG (**A**), CuPG (**B**) and ZnPG (**C**). The red arrow shows the changes in the intensity upon addition of the studied compound. The inserted graph represents the Stern–Volmer plot of the F_0_/F vs. [compound].

The investigation of interactions of metal complexes with nucleosides, nucleotides and DNA is of significance for understanding their modes of action [66]. Considering this, the binding affinity of PG, CuPG and ZnPG complexes toward ct-DNA (calf thymus DNA) was investigated by competitive studies with ethidium bromide (EthBr) and 2′-(4-hydroxyphenyl)-5-[5-(4-methylpiperazine-1-yl)benzimidazo-2-yl]-benzimidazole (Hoechst 33258; Hoe), using fluorescence emission spectroscopy (Figure 6 and Figure 7). As it was known, EthBr behaves as an intercalator between adjacent base pairs in the DNA double helix, leading to an intense fluorescence emission at 607 nm (upon excitation at 520 nm) due to the formation of the ct-DNA–EthBr system. On the other hand, Hoechst 33258 was used as a marker for minor groove binding, leading to the formation of a product having an intense fluorescent emission at 486 nm (upon excitation at 346 nm) [67,68]. Therefore, the interaction mode between a compound and ct-DNA can be determined by following the changes in the fluorescence emission spectrum of the ct-DNA–EthBr and ct-DNA–Hoe solutions upon the addition of its increasing concentration.

The emission spectra of the ct-DNA–EthBr and ct-DNA–Hoe systems were recorded in the presence and absence of an increasing concentration of the investigated compounds (Figure 6 and Figure 7). In all cases, the fluorescence intensity decreased, upon addition of the corresponding compound, suggesting an interaction with the ct-DNA. The value of *K*_A_ constants for the studied compounds indicates that they cannot successfully compete with an intercalative agent EthBr (*K*_A_ = 2 × 10^6^ M^−1^) [57] for the binding sites on ct-DNA (Table 3). Conversely, both CuPG and ZnPG can compete with Hoe, thus showing a better affinity toward minor grooves. In all cases, the calculated values of the *K*_q_ constants are larger than 2 × 10^10^ M^−1^s^−1^ (the maximum diffusion collision quenching rate constant), suggesting that the mechanism of quenching is static [57,67]. It is important to note that the values of the binding constants of PG in the presence of EthBr are in accord with previously published results [69].

**Figure 8:**
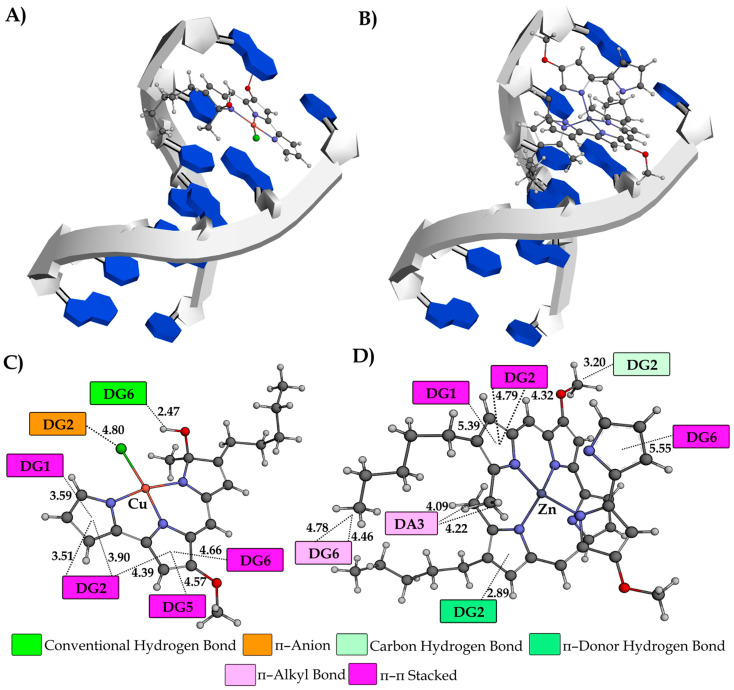
A representation of the three-dimensional structures of CuPG (**A**) and ZnPG (**B**) in their most stable conformations as intercalators within the hexanucleotide d(CGATCG)_2_ (PDB code: **1Z3F**). The sugar–phosphate backbones of the two complementary strands are shown as helically twisted white bands, with nucleobases in blue. Two-dimensional diagrams illustrate the interactions between CuPG (**C**) and ZnPG (**D**) with the nucleotides, including interatomic distances (Å) obtained from molecular docking simulations. The nucleotides are designated as DA (deoxyadenosine), DG (deoxyguanosine), DC (deoxycytidine) and DT (deoxythymidine). Different colors represent various types of interactions, as indicated in the legend. The investigated compounds are represented as gray sticks (carbon atoms), with spheres of different colors indicating specific atoms: N (blue), O (red), Cl (green), Cu (pink) and Zn (violet).

**Figure 9:**
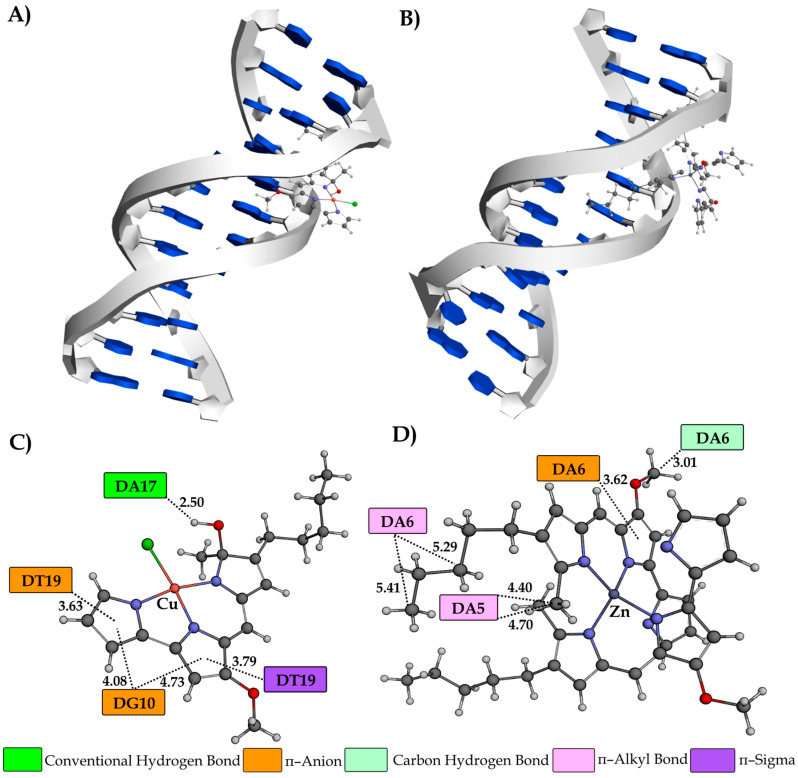
A representation of the three-dimensional structures of CuPG (**A**) and ZnPG (**B**) in their most stable conformations as minor groove binders within the dodecamer d(CGCGAATTCGCG)_2_ (PDB code: **1BNA**). The sugar–phosphate backbones of the two complementary strands are shown as helically twisted white bands, with nucleobases in blue. Two-dimensional diagrams illustrate the interactions between CuPG (**C**) and ZnPG (**D**) with the nucleotides, including interatomic distances (Å) obtained from molecular docking simulations. The nucleotides are designated as DA (deoxyadenosine), DG (deoxyguanosine), DC (deoxycytidine) and DT (deoxythymidine). Different colors represent various types of interactions, as indicated in the legend. The investigated compounds are represented as gray sticks (carbon atoms), with spheres of different colors indicating specific atoms: N (blue), O (red), Cl (green), Cu (pink) and Zn (violet).

A molecular docking study was also performed to gain a complete understanding of the binding mechanisms between the investigated compounds and DNA. The research was carried out using two distinct configurations of helically coiled antiparallel polynucleotide strands. The initial example pertains to the crystal structure of the DNA dodecamer (**1BNA**), whereas the subsequent instance concerns the crystal structure of a DNA fragment consisting of six base pairs in conjunction with an intercalating agent known as ellipticine (**1Z3F**). The quantitative results obtained from the molecular docking study are presented in Table 4, whereas Figure 8, Figure 9 and Figure S8 illustrate the most stable conformations of the investigated compounds, together with the significant interactions observed with the nucleotides in DNA molecules.

Based on the results in Table 4, the values of free binding energy (ΔG*_bind_*) and the constant of inhibition (*K_i_*), it can be inferred that both complexes demonstrate a greater propensity for binding to DNA minor grooves. These facts correlate with the experimentally obtained results. Due to the voluminous structure, the ZnPG complex shows a slightly higher affinity (−7.84 kcal mol^−1^) towards a minor groove than CuPG complex (−7.82 kcal mol^−1^). Nevertheless, PG has a greater affinity for a minor groove (−8.13 kcal mol^−1^) due to the existence of a proton of the pyrrole ring, which improves its ability to interact with nucleobases. As anticipated, the intercalation ability of the CuPG (−7.64 kcal mol^−1^) and PG (−7.59 kcal mol^−1^) compounds surpasses that of the ZnPG (−6.06 kcal mol^−1^) complex. The observed phenomenon can be attributed to the somewhat planar structures of the CuPG and PG compounds and their capacity to be effectively inserted into the ellipticine site (Figure 8A,B).

Hydrophobic interactions are the predominant interactions between the investigated compounds and DNA macromolecules (Figure 8). The dinucleotides DG1–DG2 and DG5–DG6 form *π–π* stacking interactions with the aromatic pyrrole rings of the CuPG complex. Furthermore, the dinucleotide DG1–DG2 forms several *π–π* stacking interactions with the PG at different interatomic distances (Figure S8). The nucleobase DG2 forms distinctive *π*–donor hydrogen (2.89 Å) and carbon–hydrogen (3.20 Å) interactions with the ZnPG complex. A specific *π*–donor hydrogen bond is formed between the amino group (–NH) of the DG2 nucleobase and the aromatic ring of ZnPG. The N–H·*π*–donor bond has attracted significant attention in recent times due to its prevalence in diverse biological systems and the variability in interaction energy observed across different chemical systems [70,71]. In addition, only CuPG, via a partially negative chlorine atom, forms a π–anion interaction with the aromatic ring of nucleobase DG2 (4.80 Å).

Investigation of the DNA minor groove dodecamer indicated that the most stable conformations of the investigated compounds mostly interacted with macromolecules through hydrophobic and electrostatic interactions (Figure 9).

An exception is the establishment of a conventional hydrogen bond which forms between the polar and –OH group of the CuPG and nucleobase DA17 (2.50 Å). The dinucleotide DC15–DG16 forms conventional hydrogen bonds with the polar functional groups of the pyrrole ring of PG (Figure S8). Conversely, the nucleobases DG10 (4.08 and 4.73 Å) and DT19 (3.63 Å) feature partly negative oxygen atoms that engage in the formation of a π–anion interaction with the PG aromatic rings of the CuPG complex. A comparable interaction is established between the nucleobase DA6 (3.62 Å) and the aromatic ring of the ZnPG complex. Furthermore, it should be noted that nucleobase DA6, located in the minor groove of the DNA dodecamer, forms a carbon–hydrogen (3.01 Å) and π–alkyl (5.29 and 5.41 Å) bond with the ZnPG complex.

## 3. Materials and Methods

All used chemicals and solvents were analytical-grade quality and used as received. The components of the bacteriological media were molecular biology reagent-grade quality. Peptone and tryptone were purchased from Torlak, Serbia. Agar, glycerol and anhydrous glucose were purchased from Thermo Fisher Scientific (Oxford, UK). Methanol (MeOH), ethyl acetate (EtOAc), HCl, NaCl, KCl, MgSO_4_, NaOH, Na_2_SO_4_, diethyl ether (Et_2_O), dimethyl sulfoxide (DMSO), *n*-hexane, deuterated methanol (CD_3_OD), Antifoam SE-15 A8582 aqueous emulsion with 10% active silicon, silica gel, 3-(4,5-dimethylthiazol-2-yl)-2,5-diphenyltetrazolium bromide (MTT), fetal bovine serum (FBS), penicillin, streptomycin, RPMI-1640 medium, copper(II) chloride dihydrate, zinc(II) chloride, *tert*-butanol, sodium *tert*-butoxide, dichloromethane (CH_2_Cl_2_), aluminum oxide, phosphate buffer saline (PBS), calf thymus DNA (ct-DNA), ethidium bromide (EthBr), 2′-(4-hydroxyphenyl)-5-[5-(4-methylpiperazine-1-yl)benzimidazo-2-yl]-benzimidazole (Hoechst 33258, Hoe), bovine serum albumin (BSA), eosin Y (eos Y), ibuprofen (ibu) and digitoxin (dig) were obtained from Sigma-Aldrich Chemical Co. (Steinheim, Germany).

The optimization experiments were performed in the 8-channel bioreactor Biosan RTS-8 plus (Riga, Latvia). Fermentations were conducted in a Bio4, EDF-5.4_1 bioreactor with 4.5 L volume (Biotehniskais centrs AS, Riga, Latvia). Fermentation cultures were centrifuged using Du Pont Instruments Sorvall RC-58 Refrigerated Superspeed Centrifuge, LabX Media Group, Midland, Canada. Reduced-pressure evaporation was performed using BÜCHI Rotavapor^®^ R-300 (BÜCHI Labortechnik AG, Flawil, Switzerland). PG purification was performed on silica gel (63–200 μm particles, Sigma Aldrich, Taufkirchen, Germany). Thin-layer chromatography (TLC) was conducted on silica gel 60 F_254_ (Merck, Darmstadt, Germany) and visualized under a CAMAG UV Lamp 4 at 254/366 nm (Belgrade, Serbia). Flat-bottom microtiter plates (96-well; Sarstedt, Germany) were used for the antiproliferative assay, and they were read using the Tecan Infinite 200 Pro multiplate reader (Tecan Group Ltd., Männedorf, Switzerland).

NC Technologies ESC 8020 Organic Elemental Analyzer (Waltham, MA, USA) was used to perform elemental analyses of the CuPG and ZnPG complexes for carbon, nitrogen and hydrogen. A Perkin-Elmer Spectrum Two spectrometer (Milan, Italy) was used for recording IR spectra as KBr pellets over the range of 4000–450 cm^−1^. The UV–Vis spectra were recorded on a Shimadzu double-beam spectrophotometer (Kyoto, Japan) after dissolving the copper(II) complex in DMSO over the wavelength range of 1100–200 nm and 24 and 48 h after its dissolution to monitor its solution stability. The concentration of the copper(II) complex was 1.0 × 10^−3^ M. A Jasco FP-6600 spectrophotometer (Tokyo, Japan) was used for recording the fluorescence emission spectra for the DNA/BSA interactions of PG, CuPG and ZnPG.

### 3.1. PG Production Optimization Using Waste Streams

The PG-producing strain *S. marcescens* ATCC 27117 was propagated in LB (Luria Bertani), and 2% inoculum was used to inoculate the starter culture in FB (see Table S1 for composition), as described previously [52]. For the optimization experiments, 6 different previously described waste streams with a known C/N ratio [38] were assessed for PG production: potato peels (PP), stale bread (SB), mixed waste (MW), expired yoghurt (YO), peeled boiled eggs (BE) and processed meat (PM). FB medium was used as the basis for formulating novel media based on stream-based waste, with each waste representing one group (PP, SB, MW, YO, BE, PM, Table S1), and 6 formulations of the waste media were prepared within each of the 6 groups (36 combinations in total). The starter culture (2% inoculum) was used to inoculate 7.5 mL of the waste-based media in 50 mL tubes, and the cultivation was performed at 2700 rpm for 24 h with a 1 s reverse interval spin (rsi) at 30 °C/303.15 K in the 8-channel bioreactor. All optimization experiments were performed in triplicate. Statistical analysis was performed by ANalysis Of VAriance (ANOVA) followed by Fisher’s (Least Significant Difference) post hoc test. PG production was estimated spectrophotometrically [72,73] following EtOAc/HCl extraction according to the previously established procedure [42,52].

### 3.2. PG Production and Purification in the Bioreactor

For large-scale cultivations, the starter culture (2% inoculum) was used to inoculate 3 L of the FB medium or 3 L of the optimal waste medium containing 18 g/L of PM (formulation 1, PM-1, Table S1), as described previously [52]. Pigment production was monitored spectrophotometrically, as previously described [52].

Fermentation cultures were centrifuged at 6037 rcf (relative centrifugal force) for 20 min at 4 °C/277.15 K; the cells were collected and used for PG extraction. Bacterial cells were freeze-dried and PG was extracted by vigorous shaking using 1% HCl/MeOH (20 mL per 1 g of dry cells) for 1 h. The mixture was centrifuged, the organic portion collected, dried over anh. Na_2_SO_4_ and evaporated to dryness to yield the crude PG extract. The cells were extracted 3 times following the same procedure. The crude extracts were combined and stored at 4 °C/277.15 K in the dark before chromatographic purification.

The crude extracts of PG were purified using gravitation column chromatography, as described previously [42]. The fractions containing pure PG were pulled together, evaporated under reduced pressure and stored in a solid state at −20 °C/253.15 K in the dark. The chemical structure of PG was in line with the literature values [42].

### 3.3. Synhesis of Copper(II) and Zinc(II) Complexes with PG

Copper(II) and zinc(II) complexes with PG, CuPG and ZnPG were prepared by the reaction of the corresponding metal salt with PG in the presence of sodium *tert*-butoxide in *tert*-butanol, according to the previously published method [49]. Yield: 44.0 mg (65%) for CuPG and 34.0 mg (62%) for ZnPG.

Anal. calcd for CuPG = C_20_H_24_ClCuN_3_O_2_ (MW = 437.41 g/mol): C, 57.00; H, 5.74; N, 9.97%. Found: C, 56.78; H, 5.82; N, 9.85%. IR (KBr, ν, cm^−1^): 3444br (ν(O–H)), 2985w, 2962w, 2917m (ν(C–H)), 1618vs, 1438s, 1417s, 1384m (ν(C_ar_=C_ar_) and ν(C_ar_=N)), 1262m (ν(C–O)), 803m (*γ*(C_ar_–H)). UV–Vis (DMSO, *λ*_max_, nm): 707 (ε = 3.04 × 10^2^ M^−1^cm^−1^).

Anal. calcd for ZnPG = C_40_H_48_ZnN_6_O_2_ (MW = 710.23 g mol^−1^): C, 67.64; H, 6.81; N, 11.83%. Found: C, 67.94; H, 6.65; N, 11.68%. IR (KBr, ν, cm^−1^): 3436s (ν(N–H)), 3026w (ν(C_ar_–H)), 2962w, 2925m (ν(C–H)), 1492w, 1454w, 1380w (ν(C_ar_=C_ar_) and ν(C_ar_=N)), 1262s (ν(C–O)), 803m (*γ*(C_ar_–H)).

### 3.4. Antimicrobial Activity—Disc Diffusion Assay

The following microorganisms were used to determine antimicrobial properties: three bacterial strains (*Pseudomonas aeruginosa* ATCC 1033, *Staphylococcus aureus* ATCC 25923 and *Escherichia coli* NCTC 9001) and one fungal strain (*Candida albicans* ATCC 10231) obtained from culture collections (ATCC—American Type Culture Collection, NCTC—National Collection of Type Cultures). Bacteria were grown on LB agar plates and the fungal culture on Sabouraud agar plates (SAB: 40 g/L glucose, 10 g/L peptone, 20 g/L agar) at 37 °C/310.15 K overnight. The disc assay was performed following a standard procedure [74]. The tested compounds PG, CuPG or ZnPG were dissolved in DMSO and applied to sterile discs (62.5 μg, 125 μg and 250 μg per disc), and DMSO served as a control. The plates were incubated at 37 °C/310.15 K for 24 h.

### 3.5. Antiproliferative Activity

The cytotoxicity of PG, CuPG or ZnPG against MRC-5 (lung fibroblasts) and anticancer activity against A549 (lung cancer) and HCT116 (colon cancer) cell lines, all obtained from ATCC, was measured using the standard previously described assay [75,76]. Each tested compound was added to the wells at a concentration of 0.1–500.00 μM and the treatment lasted for 48 h. Cell proliferation was determined using the MTT reduction assay in quadruplicate. The control (untreated cells) was arbitrarily set to 100%. The cell viability rate (%) was calculated as follows: (A of the treated group/A control group) × 100. The MTT reduction was followed spectrophotometrically at 540 nm using the multiplate reader.

### 3.6. BSA Binding Study

Tryptophan (TRP) fluorescence quenching experiments using BSA (5 μM) in phosphate buffer solution (PBS; pH 7.4) were performed to investigate the protein binding affinity of PG and its metal complexes. The quenching of the emission intensity of the TRP residues of BSA at 366 nm was followed after the addition of the studied compounds (up to 140 μM). The fluorescence spectra were measured in the range 285–500 nm with an excitation at 280 nm. The Stern–Volmer constants (*K_sv_*) were calculated using Equation (1) [67]:

F_0_/F = 1 + *K_q_*τ_0_[complex] = 1 + *K_sv_*[complex], (1)

where F_0_ and F represent the fluorescence intensities in the absence and presence of the investigated compounds, respectively. *K_q_* is the bimolecular quenching constant and τ_0_ (10^−8^ s) represents the lifetime of the fluorophore in the absence of the quencher. The binding constants (*K_A_*) and the number of the binding sites (*n*) can be calculated using Equation (2) [77]:

log(F_0_ − F)/F = log*K_A_* + *n* log[complex], (2)

Moreover, the competitive BSA interactions of PG, CuPG and ZnPG with eosin Y (eos Y), as a marker for site I of the subdomain IIA, ibuprofen (ibu), as a marker for site II of the subdomain IIIA, and digitoxin (dig) as a marker for site III of the subdomain IB were studied. The fluorescence emission range was between 295 and 500 nm, with the excitation wavelength at 290 nm. The solutions of BSA and site markers were mixed in equimolar amounts, and the increased concentrations of the studied compounds were added.

### 3.7. ct-DNA Binding Study

The interactions of PG, CuPG and ZnPG with ct-DNA were investigated using fluorescence emission spectroscopy. A stock solution of ct-DNA (1.43 × 10^−2^ M) was prepared in PBS buffer and its concentration was determined from the UV absorbance at 258 nm and the molar extinction coefficient, *ε* = 6.6 × 10^3^ M^−1^cm^−1^ [78]. The stock solutions of EthBr (1.01 × 10^−2^ M), Hoechst 33258 (1.0 × 10^−2^ M) and the studied compounds (1.0 × 10^−2^ M) were prepared in DMSO.

The competitive binding studies were conducted as described previously with the concentration of the tested compounds gradually increasing (up to 150 μM) [67,77].

### 3.8. Molecular Docking Study

To comprehensively evaluate the binding affinity of the studied compounds to DNA and BSA macromolecules, molecular docking studies were conducted alongside the experimental procedures using spectrofluorimetric techniques. The study employed the Autodock 4.2 program [79] in conjunction with the Lamarckian Genetic Algorithm (LGA), to assess the binding affinity of the investigated compounds towards BSA and DNA macromolecules [80]. The parameters utilized for protein-ligand rigid-flexible docking using the LGA approach were as follows: a maximum of 250,000 energy assessments, 27,000 generations and mutation and crossover rates of 0.02 and 0.8, respectively. The process of molecular docking simulation encompasses a series of consecutive steps that involve the preparation of the ligand, grid formation and identification and preparation of the BSA/DNA. CuPG and ZnPG structures [49] utilized in this investigation were sourced from the Cambridge Crystallographic Data Centre database. The structure of BSA was obtained from the RCSB Protein Data Bank (PDB) using PDB ID **4F5S** [81]. In the process of modifying the basic structure of BSA using BIOVIA Discovery Studio 4.0, only chain A was maintained, whereas chain B, heteroatoms, water molecules and residual atoms were eliminated. The search space of BSA was confined to a grid box with dimensions of 60 × 60 × 60 Å and a grid spacing of 0.375 Å. The XYZ coordinates for each site were as follows: site I (subdomain IIA) at −4.80 × 30.50 × 101.01, site II (subdomain IIIA) at 10.91 × 16.30 × 119.72 and site III (subdomain IB) at 19.86 × 33.53 × 97.92 [82]. The conformations of canonical B-DNA (PDB ID: **1BNA**) and DNA with an intercalation gap (PDB ID: **1Z3F**) were obtained from the RCSB PDB [83,84]. The grid box dimensions for the **1BNA** structure were established as 60 × 74 × 120 Å, with specific measurements of 15.81 × 21.31 × 9.88 Å and a grid spacing of 0.375 Å. The remaining docking parameters were determined according to standard approaches as described in our previous studies [85,86].

## 4. Conclusions

In this study, processed meat waste at 18 g/L (PM-1) was used as a dual source of nitrogen and carbon and was successfully integrated into the biotechnological production of PG. Additionally, the establishment of a reliable extraction (1% HCl/MeOH) and gravitation chromatography purification afforded 83.1 mg/L of PG from 3 L bacterial fermentation, which was further used for complexation with medically relevant metal ions. ZnPG and CuPG predominantly targeted site III on BSA, with ZnPG exhibiting higher affinity. PG showed superior binding affinity compared to its metal complexes, while ZnPG and CuPG demonstrated reduced cytotoxicity towards healthy cells, suggesting their potential for safer therapeutic applications. Molecular docking simulations highlighted the affinity of the tested compounds for a DNA minor groove. PG displayed the highest affinity (−8.13 kcal mol^−1^) due to its protonated pyrrole ring, enhancing interactions with nucleobases. Both CuPG and PG showed better intercalation abilities (−7.64 and −7.59 kcal mol^−1^, respectively) than ZnPG (−6.06 kcal mol^−1^), attributed to their planar structures. These findings underscore the promising therapeutic potential of PG and its metal complexes, warranting further optimization and clinical investigation.

## Figures and Tables

**Table 1:** The values of the BSA binding data for the PG and its copper(II) and zinc(II) complexes in the absence and presence of eosin Y (eos Y), ibuprofen (ibu) and digitoxin (dig) as the site markers. *K*_sv_ is the Stern–Volmer constant, *K*_q_ is the quenching rate constant, *K*_A_ is the binding constant and *n* is the number of binding sites per BSA.

	*K*_sv_ (M^−1^)	Fluorescence Quenching (%)	*K*_q_ (M^−1^s^−1^)	*K*_A_ (M^−1^)	*n*
PG–BSA	(7.48 ± 0.01) × 10^3^	40.8	7.48 × 10^11^	5.77 × 10^3^	0.97
PG–BSA–eos Y	(3.60 ± 0.01) × 10^3^	23.9	3.60 × 10^11^	3.91 × 10^4^	1.27
PG–BSA–ibu	(1.77 ± 0.02) × 10^4^	59.6	1.77 × 10^12^	8.33 × 10^4^	1.18
PG–BSA–dig	(2.35 ± 0.02) × 10^4^	61.8	2.35 × 10^12^	3.24 × 10^5^	1.31
CuPG–BSA	(2.06 ± 0.03) × 10^4^	57.9	2.06 × 10^12^	8.05 × 10^5^	1.41
CuPG–BSA–eos Y	(2.20 ± 0.04) × 10^4^	60.6	2.20 × 10^12^	4.80 × 10^5^	1.36
CuPG–BSA–ibu	(1.84 ± 0.02) × 10^4^	58.6	1.84 × 10^12^	3.93 × 10^5^	1.34
CuPG–BSA–dig	(9.85 ± 0.01) × 10^3^	57.6	9.85 × 10^11^	1.08 × 10^3^	0.72
ZnPG–BSA	(3.90 ± 0.01) × 10^3^	34.2	3.90 × 10^11^	7.14 × 10^3^	1.07
ZnPG–BSA–eos Y	(3.54 ± 0.01) × 10^3^	32.5	3.54 × 10^11^	8.39 × 10^4^	1.09
ZnPG–BSA–ibu	(4.84 ± 0.01) × 10^3^	40.2	4.84 × 10^11^	1.48 × 10^4^	1.13
ZnPG–BSA–dig	(5.69 ± 0.01) × 10^3^	35.9	5.69 × 10^11^	6.06 × 10^3^	1.00

**Table 3:** The values of the binding constants of PG, CuPG and ZnPG with ct-DNA–EthBr and ct-DNA–Hoe systems.

		*K*_sv_ (M^−1^)	Fluorescence Quenching (%)	*K*_q_ (M^−1^s^−1^)	*K*_A_ (M^−1^)	*n*
	PG	(1.00 ± 0.01) × 10^4^	47.6	1.00 × 10^12^	4.96 × 10^4^	1.18
EthBr	CuPG	(1.32 ± 0.01) × 10^3^	11.7	1.32 × 10^11^	4.95 × 10^2^	0.91
	ZnPG	(3.28 ± 0.01) × 10^3^	22.0	3.28 × 10^11^	8.86 × 10^3^	1.12
	PG	(8.33 ± 0.01) × 10^3^	44.9	8.33 × 10^11^	6.84 × 10^3^	0.98
Hoe	CuPG	(1.56 ± 0.01) × 10^3^	7.0	1.56 × 10^11^	1.37 × 10^9^	2.54
	ZnPG	(2.48 ± 0.01) × 10^3^	18.3	2.48 × 10^11^	1.89 × 10^4^	1.22

**Table 2:** The predicted thermodynamic parameters for the most favorable conformations of PG, CuPG and ZnPG in different BSA active sites (subdomain IIA, subdomain IIIA and subdomain IB) include the following: ΔG*_bind_* (binding free energy), *K_i_* (inhibition constant), ΔG*_total_* (final total internal energy), ΔG*_tor_* (torsional free energy), ΔG*_unb_* (unbound system energy), ΔG*_elec_* (electrostatic energy) and ΔG*_vdw_*_+*hbond*+*desolv*_, which combines the energies of dispersion (ΔG*_vdw_*), hydrogen bonds (ΔG*_hbond_*) and desolvation (ΔG*_desolv_*).

Conformations	ΔG*_bind_*	*K_i_* (µM)	ΔG*_inter_*	ΔG*_vdw+hbond+desolv_*	ΔG*_elec_*	ΔG*_total_*	ΔG*_tor_*	ΔG*_unb_*
PG–BSA–I	−6.75	11.23	−8.54	−8.38	−0.17	−0.90	1.79	−0.90
PG–BSA–II	−8.08	1.19	−9.87	−9.73	−0.14	−1.00	1.79	−1.00
PG–BSA–III	−8.92	0.29	−10.71	−10.64	−0.06	−0.78	1.79	−0.78
CuPG–BSA–I	−3.49	275	−5.14	−4.99	−0.15	−0.23	1.65	−0.23
CuPG–BSA–II	−5.74	60.7	−7.54	−7.45	−0.10	−0.44	1.79	−0.44
CuPG–BSA–III	−7.41	3.69	−10.43	−10.29	−0.14	−3.28	3.02	−3.28
ZnPG–BSA–I	−4.12	961	−7.13	−6.91	−0.23	−3.04	3.02	−3.04
ZnPG–BSA–II	−5.84	52	−9.12	−9.11	−0.01	−3.45	3.28	−3.45
ZnPG–BSA–III	−8.71	0.49	−10.36	−10.11	−0.25	−0.49	1.65	−0.49

**Table 4:** The predicted thermodynamic parameters for the most favorable conformations of PG, CuPG and ZnPG in DNA dodecamer (PDB: **1BNA**) and hexanucleotide DNA structure (PDB: **1Z3F**) include the following: ΔG*_bind_* (binding free energy), *K_i_* (inhibition constant), ΔG*_total_* (final total internal energy), ΔG*_tor_* (torsional free energy), ΔG*_unb_* (unbound system energy), ΔG*_elec_* (electrostatic energy) and ΔG*_vdw_*_+*hbond*+*desolv*_, which combines the energies of dispersion (ΔG*_vdw_*), hydrogen bonds (ΔG*_hbond_*) and desolvation (ΔG*_desolv_*).

Conformations	ΔG*_bind_*	*K_i_* (µM)	ΔG*_inter_*	ΔG*_vdw+hbond+desolv_*	ΔG*_elec_*	ΔG*_total_*	ΔG*_tor_*	ΔG*_unb_*
DNA (**1Z3F**)–PG	−7.59	2.73	−9.38	−9.23	−0.15	−0.94	1.79	−0.94
DNA (**1BNA**)–PG	−8.13	1.10	−9.91	−9.68	−0.23	−1.33	1.79	−1.33
DNA (**1Z3F**)–CuPG	−7.64	2.49	−9.29	−9.04	−0.25	−0.54	1.65	−0.54
DNA (**1BNA**)–CuPG	−7.82	1.85	−9.47	−9.52	0.05	−0.29	1.65	−0.29
DNA (**1Z3F**)–ZnPG	−6.09	34.34	−9.11	−8.95	−0.16	−3.08	3.02	−3.08
DNA (**1BNA**)–ZnPG	−7.84	1.79	−10.86	−10.81	−0.05	−2.67	3.02	−2.67

## Data Availability

The original contributions presented in the study are included in the article/Supplementary Materials; further inquiries can be directed to the corresponding authors.

## Supplementary Materials

The following supporting information can be downloaded at: https://www.mdpi.com/article/10.3390/ijms25158395/s1.
