# Supplementary material for: Copper(II) and Zinc(II) Complexes with Bacterial Prodigiosin Are Targeting Site III of Bovine Serum Albumin and Acting as DNA Minor Groove Binders
Source: Int J Mol Sci. 2024 Aug 1;25(15):8395. doi: 10.3390/ijms25158395 (PMC11313072; doi:10.3390/ijms25158395)
Supplement: Supplementary file 1 [file ijms-25-08395-s001.zip › ijms-3093555-supplementary.pdf]

# Copper(II) and Zinc(II) Complexes with Bacterial Prodigiosin are Targeting Site III of Bovine Serum Albumin and Acting as DNA Minor Groove Binders

**Lena Pantelic [1], Sanja Skaro Bogojevic [1], Tina P. Andrejević [2], Bojana V. Pantović [2], Violeta R. Marković [2], Darko P. Ašanin [3], Žiko Milanović [3], Tatjana Ilic-Tomic [1], Jasmina Nikodinovic-Runic [1], Biljana Đ. Glišić [2,*] and Jelena Lazic [1,*]**

[1] Institute of Molecular Genetics and Genetic Engineering, University of Belgrade, Vojvode Stepe 444a, 11000 Belgrade, Serbia; lpantelic@imgge.bg.ac.rs (L.P.); sanja.bogojevic@imgge.bg.ac.rs (S.S.B.); tatjanait@imgge.bg.ac.rs (T.I.-T.); jasmina.nikodinovic@imgge.bg.ac.rs (J.N.-R.)

[2] Department of Chemistry, Faculty of Science, University of Kragujevac, Radoja Domanovića 12, 34000 Kragujevac, Serbia; tina.andrejevic@pmf.kg.ac.rs (T.P.A.); bojana.pantovic@pmf.kg.ac.rs (B.V.P.); violeta.markovic@pmf.kg.ac.rs (V.R.M.)

[3] Department of Science, Institute for Information Technologies Kragujevac, University of Kragujevac, Jovana Cvijića bb, 34000 Kragujevac, Serbia; darko.asanin@uni.kg.ac.rs (D.P.A.); ziko.milanovic@uni.kg.ac.rs (Ž.M.)

* Correspondence: biljana.glisic@pmf.kg.ac.rs (B.Đ.G.); jelena_lazic@imgge.bg.ac.rs (J.L.).

## Table of Contents

**Table S1**........ 2
**Figure S1.** ........ 3
**Table S2**........ 4
**Figure S2** ........ 5
**Figure S3.** ........ 6
**Figure S4.** ........ 7
**Figure S5.** ........ 8
**Figure S6.** ........ 9
**Figure S7.** ........ 10
**Figure S8.** ........ 11

**Table S1.** The formulations (1-6) of the novel waste-based media. Fermentation Broth (FB) medium was used as the basis. Each of the 6 waste streams represents one group: potato peel (PP), stale bread (SB), mixed waste (MW), yogurt (YO), peeled boiled eggs (BE), processed meat (PM).

| **Formulation** | **Waste* (g/L)** | **Glycerol (v/v)** | **Peptone (g/L)** | **NaCl (g/L)** | **KCl (g/L)** | **$MgSO_4$ (g/L)** |
|---|---|---|---|---|---|---|
| FB (control) | / | 0.30% | 15 | 3 | 2 | 2 |
| 1 | 18 | / | / | / | / | / |
| 2 | 18 | / | / | 3 | 2 | 2 |
| 3 | 15 | 0.30% | / | 3 | 2 | 2 |
| 4 | 15 | / | 15 | 3 | 2 | 2 |
| 5 | 9 | 0.15% | 7.5 | 3 | 2 | 2 |
| 6 | 3.5 | 0.24% | 12 | 3 | 2 | 2 |

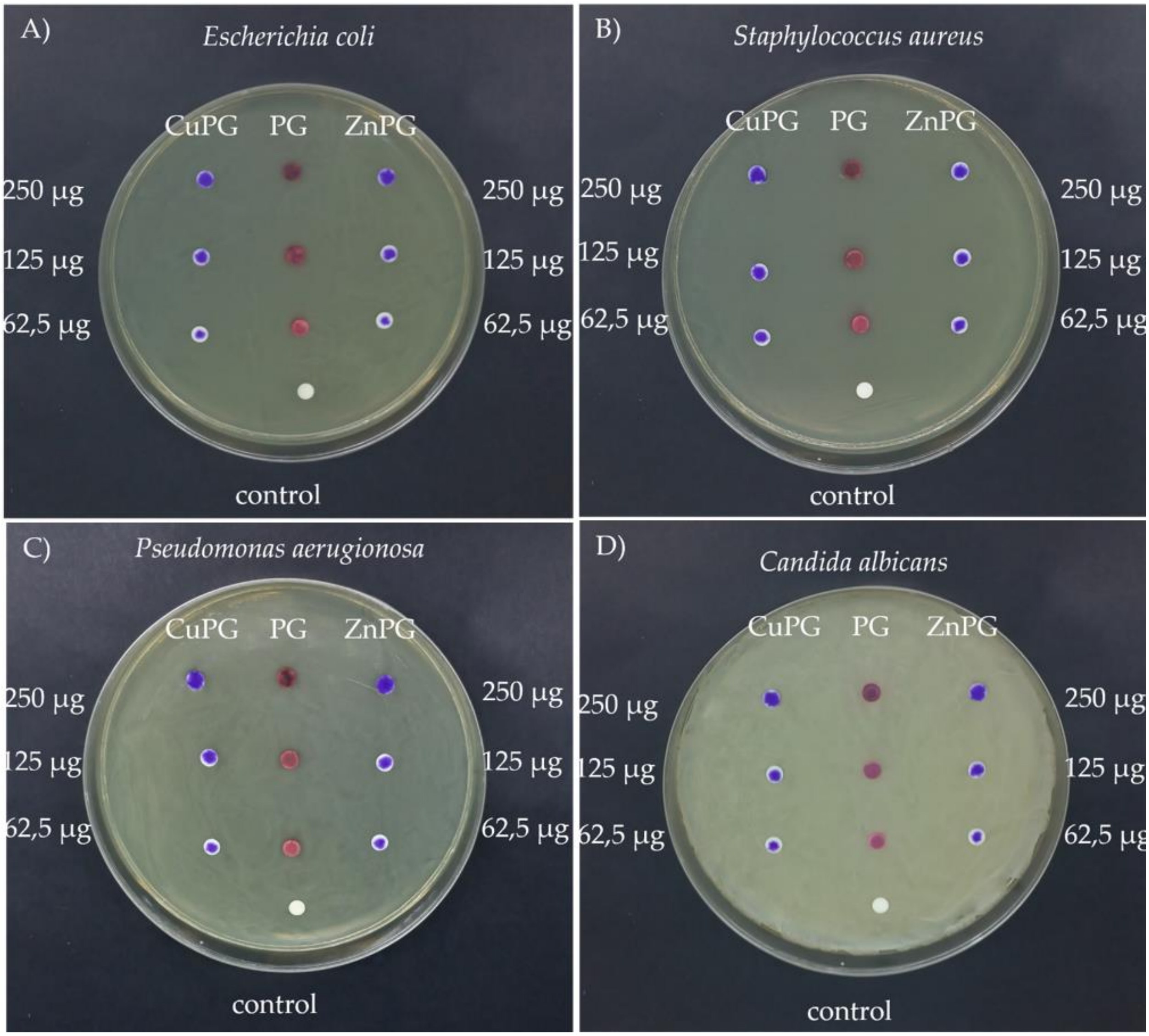


**Figure S1.** Antimicrobial evaluation of PG, CuPG and ZnPG by disc diffusion assay using three different concentrations (250 µg, 125 µg and 62.5 µg/disc): antibacterial activity against A) *E. coli*, B) *P. aeruginosa* and C) *S. aureus*; D) antifungal activity against *C. albicans*.

**Table S2.** Antiproliferative activity ($IC_{50}$, μM) of PG, CuPG and ZnPG after 48 h treatment.

| Compound | MRC-5 | A549 | HCT116 |
|---|---|---|---|
| PG | 3.71 ± 0.12 | 4.02 ± 0.06 | 2.16 ± 0.06 |
| CuPG | 91.45 ± 0.25 | 80.02 ± 0.48 | 114.31 ± 0.30 |
| ZnPG | 42.24 ± 0.10 | 105.60 ± 0.27 | 35.20 ± 0.24 |

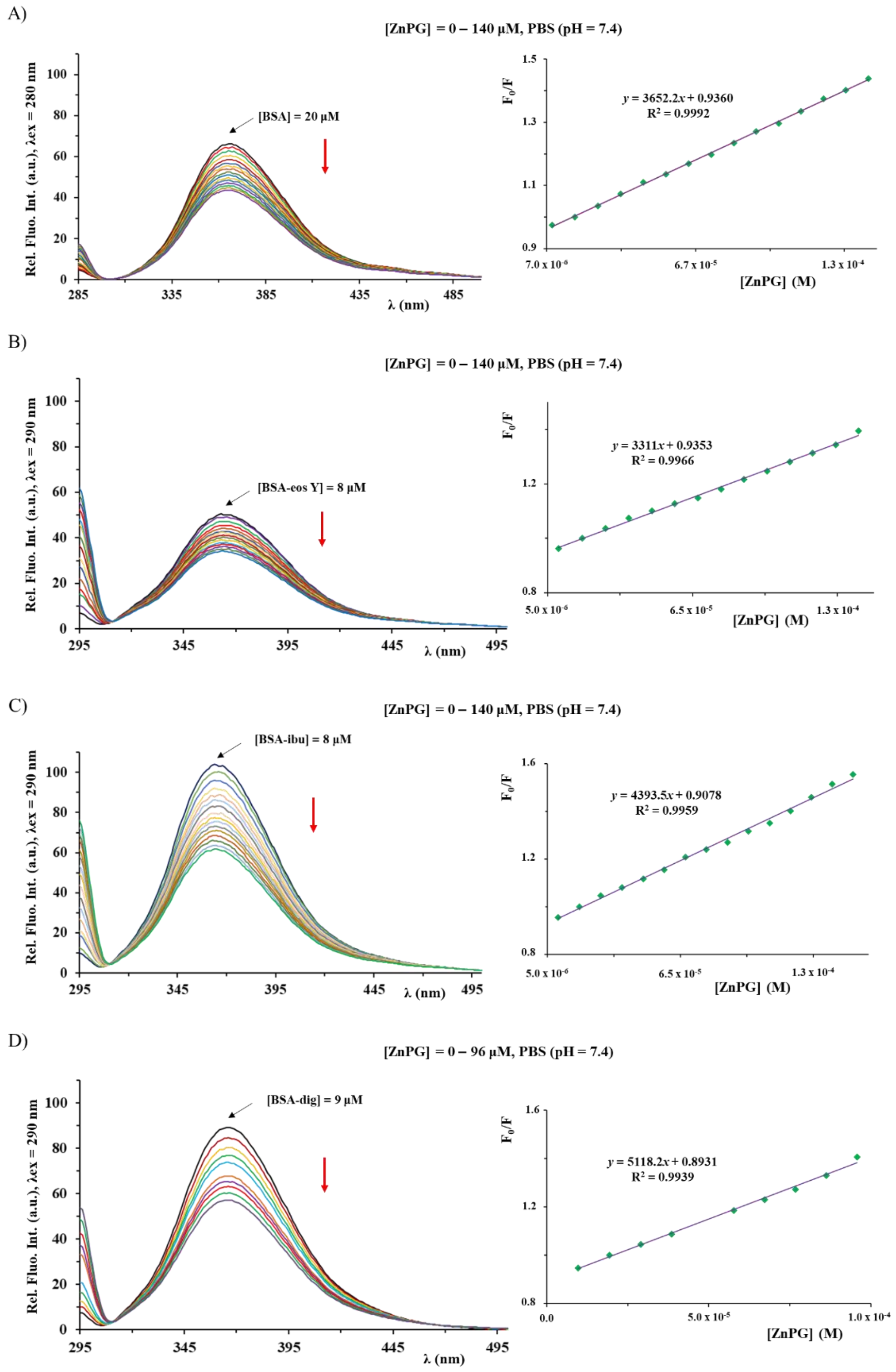


**Figure S2.** BSA fluorescence emission spectra in the presence of an increasing concentration of ZnPG (A) and in presence of the site markers (B – D). The red arrow shows the changes of the intensity after the addition of the complex. The inserted graph shows the $F_0/F$ dependence of complex concentration.

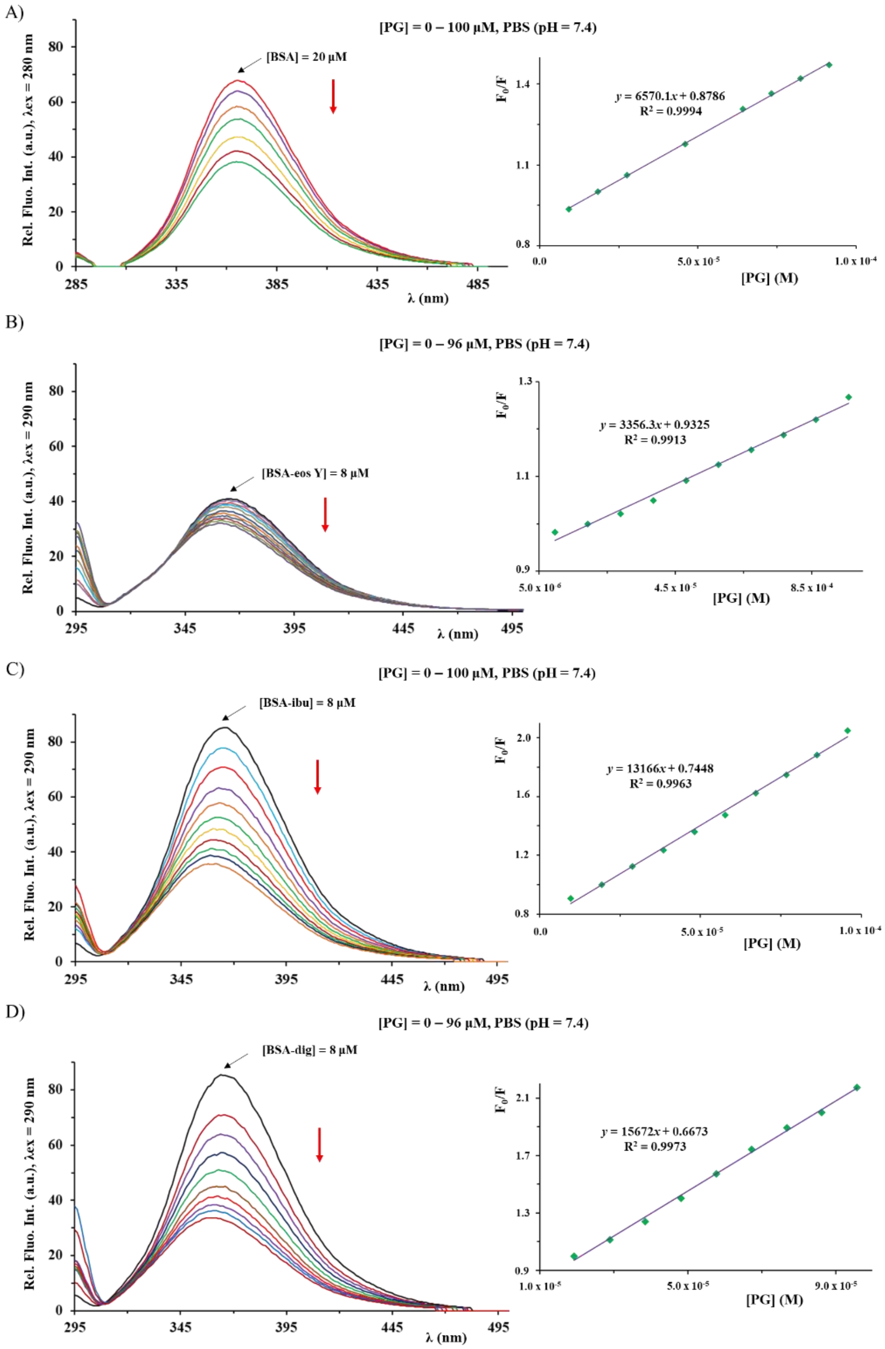


**Figure S3.** BSA fluorescence emission spectra in the presence of an increasing concentration of PG (A) and in presence of the site markers (B – D). The red arrow shows the changes of the intensity after the addition of the complex. The inserted graph shows the $F_0/F$ dependence of complex concentration.

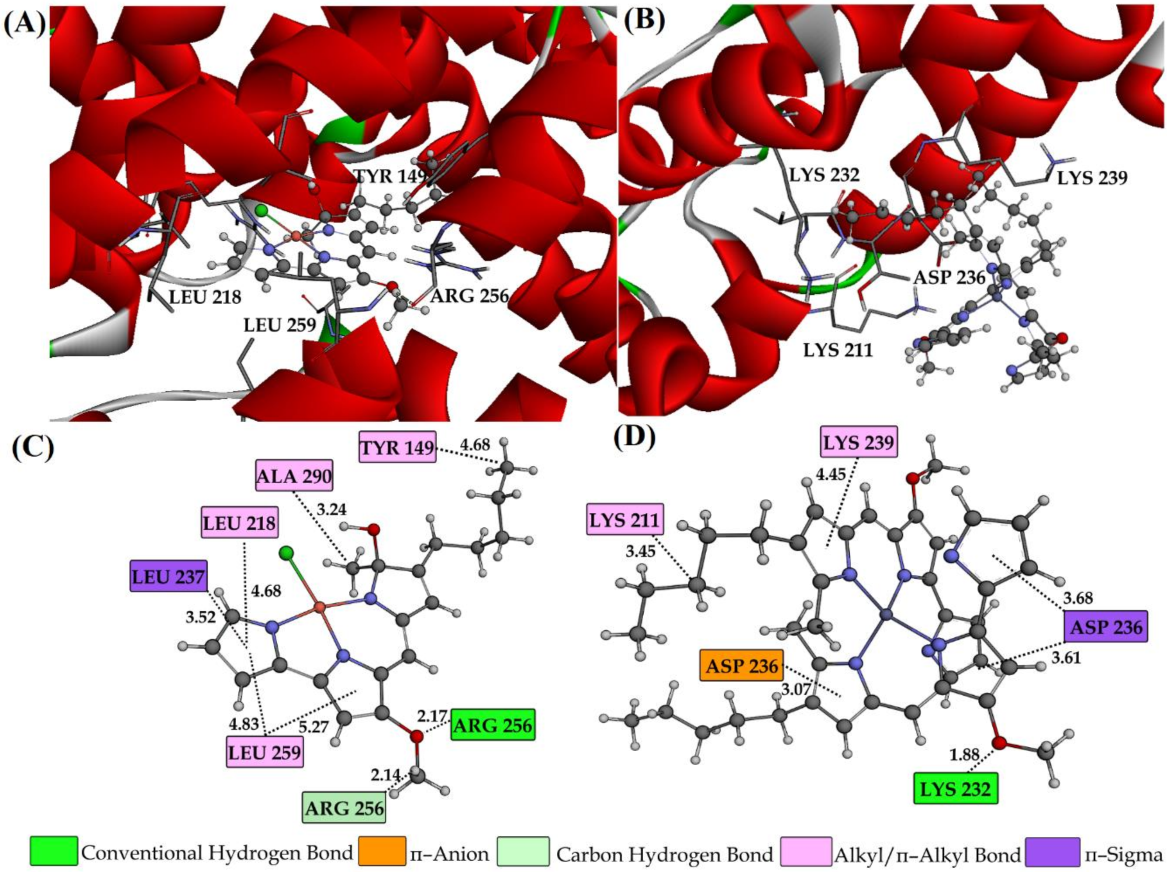


**Figure S4.** Three-dimensional representation of the most stable favorable docking position of CuPG (A) and ZnPG (B) in subdomain IIA (active site I) of BSA (PDB code: **4F5S**). Investigated complexes are represented as grey sticks (carbon atoms). Different colors on the sphere indicate different atoms: N – blue, O – red, Cl – green, Cu – pink, Zn – violet. For clarity, the remaining part of the protein structure has been excluded. Two-dimensional representation of the interactions between the CuPG (C) and ZnPG (D) and BSA with interatomic distance (Å) obtained after molecular docking study. Various colors are indicative of distinct types of interactions (legend).

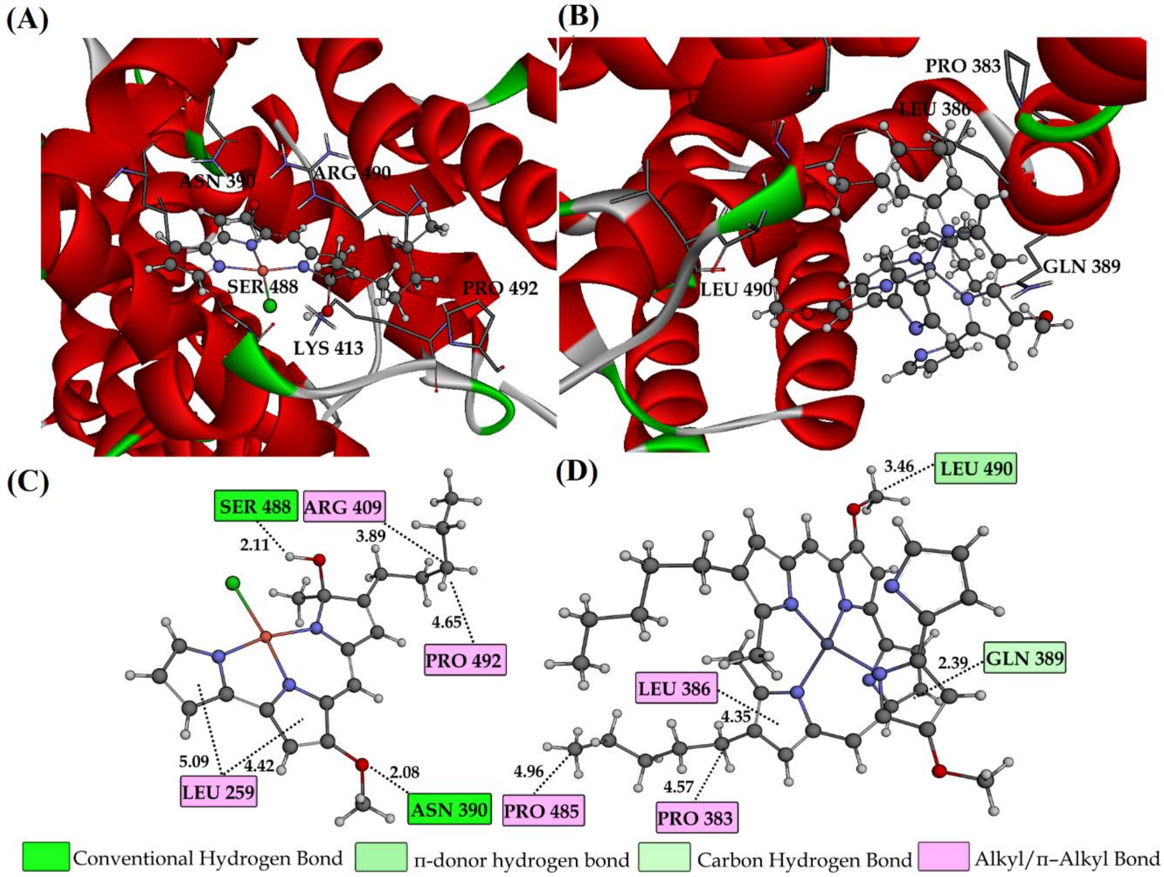


**Figure S5.** Three-dimensional representation of the most stable favorable docking position of CuPG (A) and ZnPG (B) in subdomain IIIA (active site II) of BSA (PDB code: **4F5S**). Investigated complexes are represented as grey sticks (carbon atoms). Different colors on the sphere indicate different atoms: N – blue, O – red, Cl – green, Cu – pink, Zn – violet. For clarity, the remaining part of the protein structure has been excluded. Two-dimensional representation of the interactions between the CuPG (C) and ZnPG (D) and BSA with interatomic distance (Å) obtained after molecular docking study. Various colors are indicative of distinct types of interactions (legend).

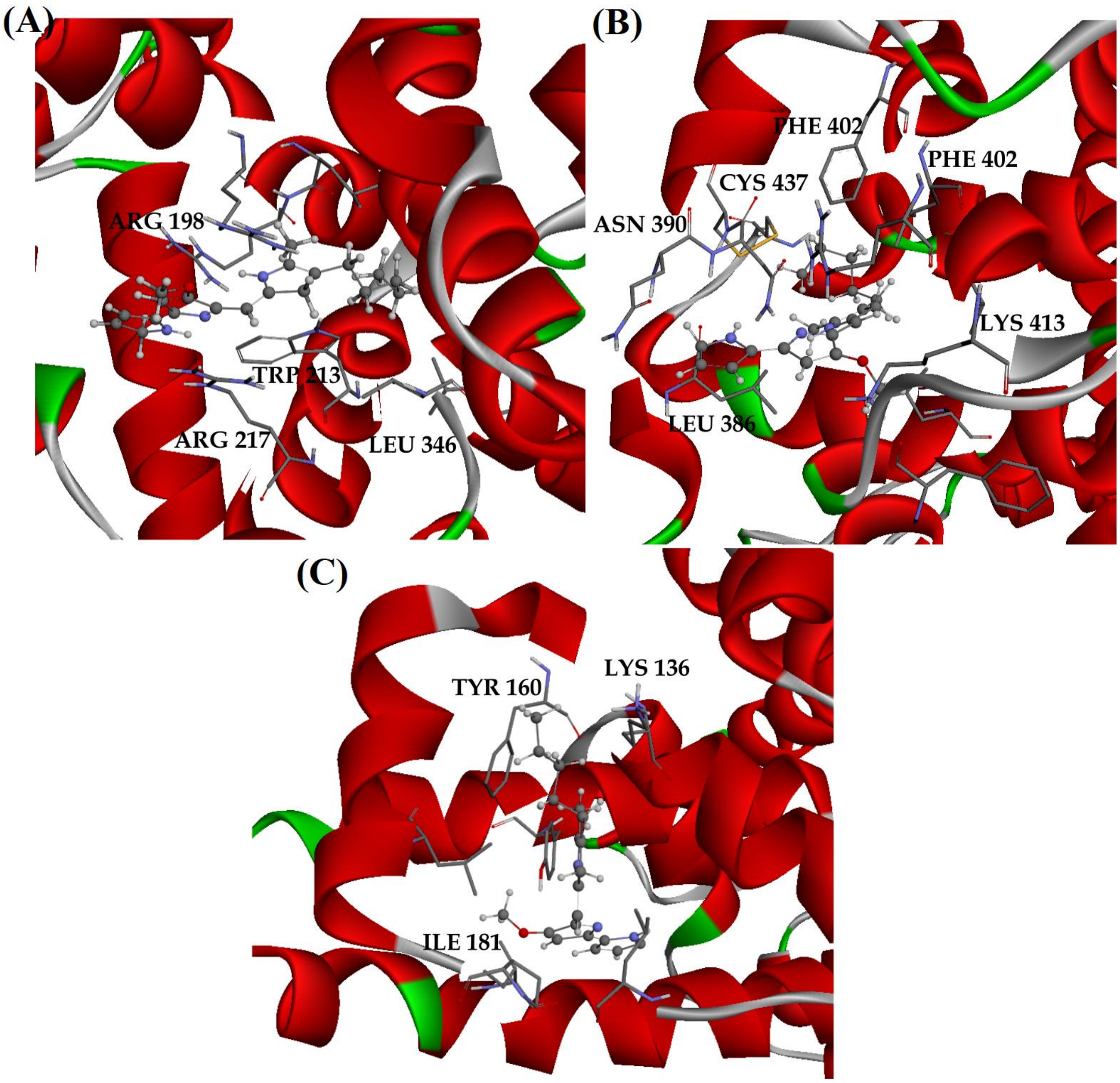


**Figure S6.** Three-dimensional representation of the most stable favorable docking position of PG in subdomain IA (active site I) (A), subdomain IIIA (active site II) (B) and subdomain IB (active site III) (C) of BSA (PDB code: **4F5S**). The investigated compound is represented as grey sticks (carbon atoms). Different colors on the sphere indicate different atoms: N – blue, O – red, Cl – green. For clarity, the remaining part of the protein structure has been excluded.

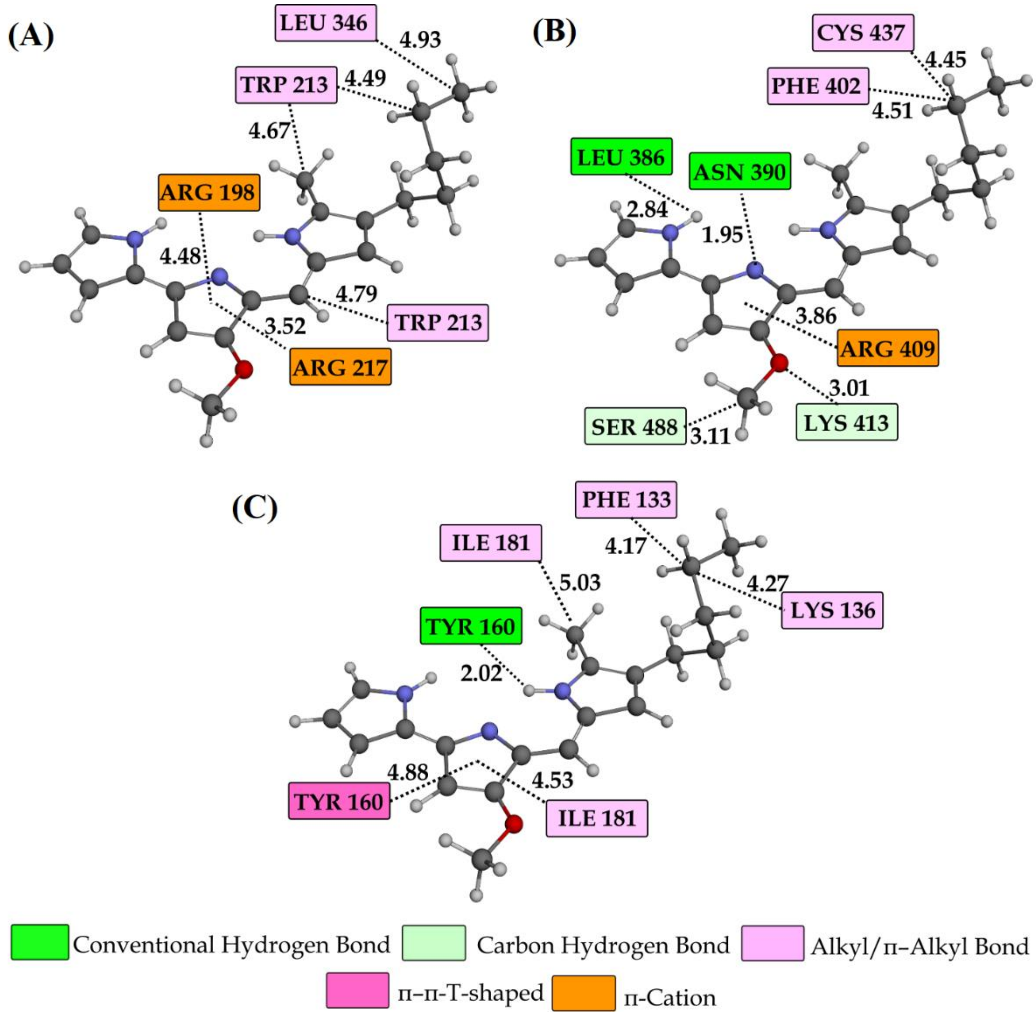


**Figure S7.** Two-dimensional representation of the interactions between the PG and BSA (PDB code: **4F5S**) amino acid residues with interatomic distance (Å) obtained after molecular docking study. Various colors are indicative of distinct types of interactions (legend).

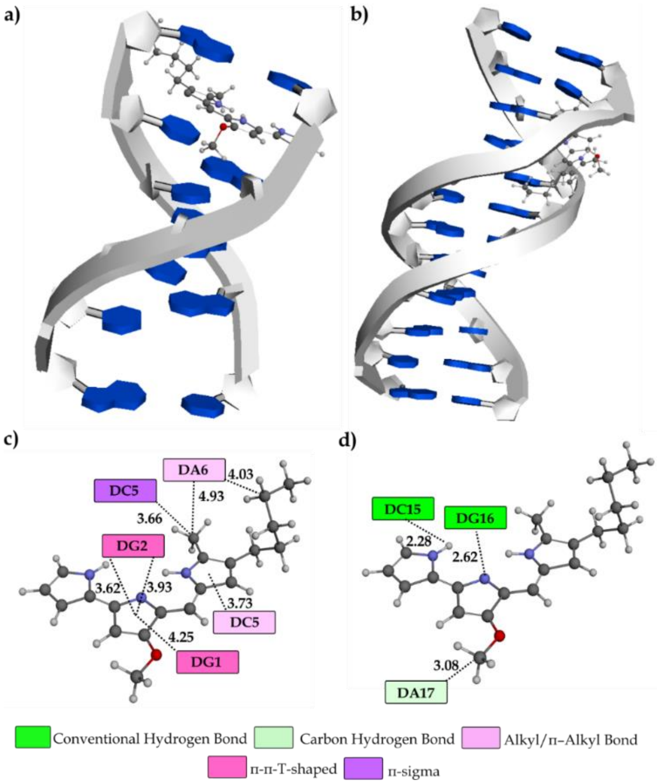


**Figure S8.** Three-dimensional representation of the most stable conformations of PG in the hexanucleotide d(CGATCG)$_2$ (PDB code: **1Z3F**) (A) and dodecamer d(CGCGAATTCGCG)$_2$ (PDB code: **1BNA**) (B). The depiction illustrates the sugar-phosphate backbones of the two complimentary chains as helically twisted white bands, with the nucleobases represented in blue. Two-dimensional representation of the PG in the hexanucleotide d(CGATCG)$_2$ (PDB code: **1Z3F**) (C) and dodecamer d(CGCGAATTCGCG)$_2$ (PDB code: **1BNA**) (D) with interatomic distance obtained after molecular docking simulation (DA = deoxyadenosine; DG = deoxyguanosine; DC = deoxycytidine; DT = deoxythymidine). Different colors indicate different types of interactions (legend). Different colors on the sphere indicate different atoms: N – blue, O – red, Cl – green.
